# Regulation of Life & Death by REGγ

**DOI:** 10.3390/cells11152281

**Published:** 2022-07-23

**Authors:** Keaton E. Funderburk, Jungseog Kang, Henry J. Li

**Affiliations:** School of Arts and Sciences, New York University Shanghai, 1555 Century Avenue Pudong, Shanghai 200135, China; kef399@nyu.edu (K.E.F.); jungseog.kang@nyu.edu (J.K.)

**Keywords:** REGγ, proteasome, tumor suppressor, regulation

## Abstract

REGγ, a proteasome activator belonging to the 11S (otherwise known as REG, PA28, or PSME) proteasome activator family, is widely present in many eukaryotes. By binding to the 20S catalytic core particle, REGγ acts as a molecular sieve to selectively target proteins for degradation in an ATP- and ubiquitin-independent manner. This non-canonical proteasome pathway directly regulates seemingly unrelated cellular processes including cell growth and proliferation, apoptosis, DNA damage response, immune response, and metabolism. By affecting different pathways, REGγ plays a vital role in the regulation of cellular life and death through the maintenance of protein homeostasis. As a promoter of cellular growth and a key regulator of several tumor suppressors, many recent studies have linked REGγ overexpression with tumor formation and suggested the REGγ-proteasome as a potential target of new cancer-drug development. This review will present an overview of the major functions of REGγ as it relates to the regulation of cellular life and death, along with new mechanistic insights into the regulation of REGγ.

## 1. Introduction

Cellular protein levels are maintained through constant translation and degradation, and proper regulation of this homeostatic equilibrium throughout the cell cycle is essential for cellular processes such as cell-cycle progression and adaptation [1]. The 20S proteasome is a major structure to degrade proteins in the cell. It is a cylindrical multisubunit protease that has a well-defined structure with an internal lumen containing enzymatic sites associated with chymotrypsin-like, trypsin-like, and caspase-like activity [2]. To regulate the proteasome, a proteasome activator such as REG binds to the 20S catalytic core particle and selectively enhances the degradation of target proteins. In humans and other jawed vertebrates, three paralogs of the 11S proteasome activator family exist: REGα, REGβ, and REGγ. Unlike REGα and REGβ, which are believed to have evolved more recently and are primarily involved in MHC class 1 antigen presentation, REGγ is involved in the regulation of an array of cellular processes such as cell growth and proliferation, apoptosis, DNA damage response, and chromatin organization. Recently, the structure and distribution of REGγ as they relate to those of REGα and REGβ have been analyzed, with attention being given to the mechanism by which these proteasome activators regulate the 20S proteasome [3]. Structurally, REGγ forms homoheptameric rings that attach to both ends of the 20S tube structure. This ring structure serves as a regulatory cap that controls access of target proteins into the lumen of the 20S proteasome for their degradation [4]. Therefore, REGγ carries out its primary cellular function of stimulating proteasomal degradation of target peptides and proteins by controlling substrate access. 

Interestingly, REGγ has also been shown to play additional roles when not in complex with 20S. One early study using immunofluorescent labeling found that REGγ was present in autophagosome-resembling perinuclear inclusion bodies, despite the absence of the proteasome [5]. Following this, REGγ was shown to localize on chromosomes during the telophase to regulate spindle integrity independently of the 20S proteasome, whereby REGγ appears to increase spindle strength [6]. REGγ has also been found to regulate chromatin compaction in a manner not dependent on binding with 20S since its depletion appears to correlate with chromatin decompaction, likely through the maintenance of histone modifications [7]. With label-free protein quantification having revealed that less than 5% of the total amount of REGγ within the cell is likely to be bound to 20S at any given time, proteasome-independent roles such as these can be anticipated [8].

**Figure 1:**
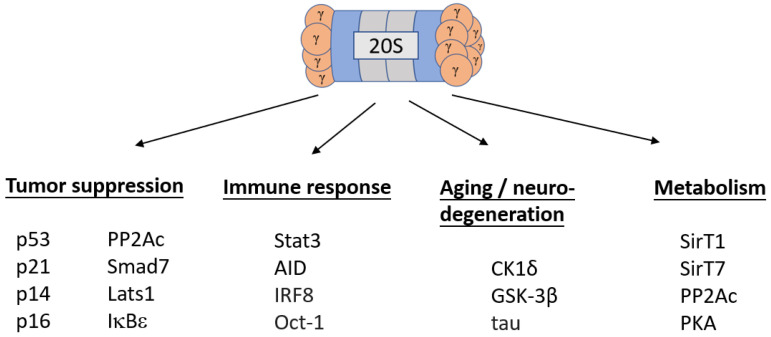
Downstream targets of the REGγ-proteasome. From tumor suppressors and immune response factors to proteins involved in aging and metabolism, REGγ regulates a broad array of biological functions via the degradation of key proteins and peptides. For abbreviations, extended names are as follows: Protein Phosphatase 2 A catalytic subunit (PP2Ac), Mothers against decapentaplegic 7 (Smad7), Large Tumor Suppressor Kinase 1 (Lats1), Inhibitor of κB epsilon (IκBε), Signal Transducer and Activator of Transcription 3 (Stat3), Activation-Induced Deaminase (AID), Interferon Regulatory Factor 8 (IRF8), Casein Kinase 1 sigma (CK1δ), Glycogen Synthase Kinase-3 beta (GSK-3β), Sirtuin 1 (SirT1), Sirtuin 7 (SirT7), and Protein Kinase A (PKA).

Since the first discovery of the steroid receptor coactivator-3 (SRC-3/AIB1) as an intact mammalian substrate of the REGγ-proteasome in 2006 [9], many direct or indirect targets of REGγ have been additionally identified, suggesting its important functions in cell physiology (Figure 1). It is predominantly localized to the nucleus and downregulates many tumor suppressors, including p53 [10], p21 [11], p14 [12], p16 [12], Lats1 [13], PP2Ac [14], IκBε [15], and Smad7 [16,17], indicating its oncogenic properties. Factors involved in immune responses are also found as REGγ targets, including signal transducer and activator of transcription 3 (Stat3) [14], activation-induced deaminase (AID) [18], interferon regulatory factor 8 (IRF8) [19], and Oct-1 [20]. Likewise, some key proteins involved in metabolism that are also degraded by REGγ include SirT1 [21], SirT7 [22], and protein kinase A (PKA) [23]. Intriguingly, additional REGγ targets include factors involved in neurodegenerative diseases such as tauopathies or Alzheimer’s disease, and the decline of REGγ was shown to be associated with the acceleration of aging-related brain disorders [24]. Our unpublished results that REGγ instructs the direct degradation of tau protein in vitro and in vivo further supports previous reports of REGγ’s involvement in tauopathies. Thus, REGγ regulates a myriad of important biological processes by both directly and indirectly changing the cellular concentrations of a wide array of proteins.

Significant advances in REGγ-related research have been made since the early discovery of the molecular functions of the REGγ-proteasome. Yet, the major coherent biological functions of REGγ via the regulation of protein degradation in cellular processes still remain to be fully encapsulated. Thus, this review will summarize the current state of knowledge regarding REGγ’s functions in the regulation of cell life and death, discuss newly discovered pathways for the regulation of REGγ, and advise future directions of research.

## 2. Regulation of Life

REGγ is capable of regulating cell viability by facilitating cell proliferation, keeping the proper balance of energy metabolism, and indirectly regulating key proteins involved in spermatogenesis, such as PLZF. The proper expression of functional REGγ is necessary for the homeostasis of the cellular proteome, whereas overexpression has been linked with excessive cellular growth and several types of cancer.

### 2.1. Cell Growth and Proliferation

REGγ is a key regulator of cellular growth and proliferation. In two of the early studies investigating the effects of murine REGγ deficiency, researchers identified that REGγ (−/−) mice developed more slowly and reached a smaller overall body size compared to REGγ (+/−) and REGγ (+/+) counterparts. Flow cytometric analysis further showed that REGγ (−/−) mice had an increased number of cells in the G1 phase and fewer cells in the S and G2/M phases of the cell cycle [25,26]. Depletion of REGγ through RNA interference in a Drosophila cell line also resulted in partial arrests of G_1_/S cell cycle transitions, confirming the results of the mouse model. A sequence search of the REGγ promoter region identified transcription regulatory elements which often appear in the promoter regions of genes involved in DNA replication and cell cycle progression, providing further evidence for REGγ’s role in cell cycle regulation [27]. Consistent with its important role in cell cycle regulation, excessive expression of REGγ was correlated with tumor development in several types of cancers, including colon cancer, lung cancer, liver cancer, and squamous cell carcinoma [28]. 

In the pioneering studies aimed at identifying direct targets of the REGγ-proteasome, it was found that degradation of unbound p21 was promoted in the presence of REGγ [11,12]. Following this, several studies clearly demonstrated links between REGγ deficiency and the upregulation of cell or tissue specific tumor-suppressor proteins. One study discovered that REGγ downregulates p53 via the casein kinase (CK) 1 pathway. CK1δ inactivates murine double minute (Mdm) 2, which stabilizes p53 because Mdm2 stimulates the ubiquitination of p53 for its degradation. By targeting CK1δ, a pathway of REGγ-CK1δ-Mdm2-p53 could be established [10]. Another recent study of pancreatic ductal adenocarcinoma showed that inhibition of REGγ-mediated SirT7 degradation by O-GlcNAc transferase resulted in repression of tumor suppressor genes and promotion of cancer cells [29]. Furthermore, Wang et al. found that REGγ was overexpressed in over 60% of the 172 colorectal cancer specimens that were analyzed, correlating with increased Yes-Associated Protein (YAP) and p65 levels. REGγ depletion significantly diminished tumor growth, but constitutively active YAP was able to overcome this effect, thus indicating YAP’s role as a mediator between REGγ and colorectal cancer development. Mechanistic analysis disclosed that in these cancer cells, REGγ directly interacted with Lats1 of the Hippo signaling pathway to promote its degradation, thus upregulating YAP activity and gene transcription [13]. As it relates to the regulation of other signaling pathways, one study on a human myeloma cell line found that silencing REGγ also downregulated the NF-κB signal pathway by preventing degradation of IκBε, causing the inhibition of cell proliferation and promotion of apoptosis [15]. REGγ mediated degradation of GSK-3β, a tumor suppressor and regulator for Wnt/β-catenin signaling, provides additional examples of upregulation of oncogenic pathways [30,31]. Moreover, a recent study on intestinal stem cells treated with radiation found that REGγ enhances the transcriptional activation of leucine-rich repeat-containing G protein-coupled receptor 5 (Lgr5), an intestinal stem-cell marker involved in regeneration, via the potentiation of both Wnt and Hippo signaling [32]. Thus, given its capacity to regulate cell cycle progression and its ability to downregulate an array of tumor suppressor proteins, the expression of REGγ significantly contributes to cell growth and proliferation.

### 2.2. Energy Metabolism

REGγ also possesses the capacity to regulate energy metabolism, which is essential for cell growth [33]. In one early study, mice deficient in REGγ exhibited higher autophagy and protection against steatosis in the liver when fed with a high fat diet, indicating cross-talk between REGγ and the autophagy system in the regulation of lipid homeostasis. Mechanistically, REGγ was found to bind to and degrade SirT1, a deacetylase that regulates autophagy and metabolism, preventing it from deacetylating, thus activating autophagy-related proteins [21]. More recently, the REGγ-proteasome has been found to act as a promoter of glycolysis in liver cancer by upregulating mTOR complex 1 (mTORC1) signaling. Mechanistically, the REGγ-proteasome degrades PP2Ac, the phosphatase that dephosphorylates the mTORC1 inhibitor PRAS40, to prevent PRAS40 from binding to Raptor and downregulating mTORC1 signaling. Thus, an REGγ-PP2Ac-PRAS40 regulatory axis could be established, further indicating REGγ’s role in regulating glycolytic signaling [34]. In another study investigating REGγ’s role in energy homeostasis, REGγ was found to be upregulated during times of starvation, whereas the ubiquitin-dependent proteasome system (UPS) was not. Due to the high amount of energy required for ubiquitin-dependent protein degradation, upregulating REGγ would preserve cellular energy levels during times of starvation since REGγ-proteasomal degradation does not require ATP. Furthermore, REGγ was found to repress rDNA transcription during times of cellular energy deficit to reduce the large demand of intracellular energy for this process. To do so, the REGγ-proteasome targets SirT7, an rDNA transcriptional activator, for degradation. Importantly, researchers also found that REGγ deficiency sensitized cells to starvation-induced apoptosis under glucose-deprived conditions in a manner not dependent on p53, indicating REGγ inhibition as a potential strategy for tumor-starving cancer therapies [22]. Hence, REGγ helps to maintain cellular energy levels in multiple cellular contexts and regulates signaling involved in energy metabolism.

### 2.3. Reproduction

Properly functioning REGγ is necessary for successful reproduction due to its role in regulating spermatogenesis. PA200, another proteasome activator that binds with 20S, works with REGγ to regulate male fertility. Even though most of the sperm cells in REGγ/PA200 double knockout mice exhibited relatively normal morphological appearances, the double knockout mice were completely infertile due to a reduction in spermatozoa mobility. To explain this phenomenon, researchers conducted quantitative analyses of protein expression levels in the double knockout mice and found that several proteins involved in oxidative damage response were being upregulated. This indicates the potential role of proteasome activators in oxidative damage response, along with the relationship between this role and male fertility [35]. Further pointing to REGγ’s important role in reproduction, ablation of murine REGγ led to increased expression of p53 that transcriptionally repress promyelocytic leukemia zinc finger protein (PLZF), a protein necessary for male fertility [36]. Haplodeficiency of p53 partially rescued the defects in spermatogenesis in REGγ KO mice by displaying a subfertile phenotype which is most likely due to the presence of PA200 [37]. Hence, REGγ can be established as an important regulator of reproductive function.

REGγ is essential for maintaining the viability of eukaryotic cells. REGγ does so by regulating important cell proliferation signaling cascades such as the Wnt/β-catenin, the NF-κB, the mTORC1 and the Hippo pathways. Additionally, it downregulates tumor suppressor proteins to fine-control cellular homeostasis, and its hyper-activation directly stimulates tumorigenesis. Furthermore, REGγ’s capacity to maintain balanced cellular energy levels by regulating energy metabolism makes it an important element for maintaining cellular fitness. These capabilities along with its necessary role as a regulator of male fertility through the downregulation of p53 and the enhancement of spermatozoa mobility establish REGγ as an important regulator of cell life.

## 3. Regulation of Death

In contrast to its role as a regulator of various processes involved in maintaining cell life, REGγ also contributes to cell death upon its loss of function. As shown previously, overexpression of REGγ often contributes to tumor formation and excessive growth, but underexpression has also been linked with increased levels of apoptosis, aging and neurodegenerative disorders, reduced spindle integrity, and slower DNA damage repair.

### 3.1. Apoptosis

One of the most notable functions of REGγ as it relates to the regulation of death is its capacity to cause apoptosis upon attenuation. Several studies have found a correlation between reduced levels of REGγ and increased levels of apoptosis [15,25,38,39,40]. The fact that depletion of REGγ sensitized cells to stress-induced apoptosis is likely due to the deregulation of p53 [41]. Mechanistically, p53 is known to stimulate apoptosis via transcriptional upregulation of pro-apoptotic proteins PUMA and NOXA [42]. Thus, reduction of REGγ contributes to increased levels of p53 as well as the pro-apoptotic factors that lead to apoptosis. Additionally, REGγ is a known substrate of caspases 3 and 7, so these caspases attenuate REGγ and further reinforce apoptosis. Interestingly, REGγ proteins inhibited caspase activity in vitro, indicating a mutually inhibitive relationship [43]. Thus, a loss of REGγ could upregulate apoptosis due to a lack of caspase inhibition. Furthermore, in a study on starvation-induced proteasome assemblies, inhibition of either REGγ or RAD23B, a proteasome shuttling factor, in amino acid-depleted cells prevented p53/NOXA upregulation and apoptosis [44]. This seems to indicate that the down-regulatory effect that REGγ has on apoptosis under normal conditions is reversed in times of cellular energy deficit. This data coincides with REGγ’s role in preserving cellular energy levels during starvation since the REGγ-proteasome appears to contribute to tissue fitness under such conditions. Hence, through the regulation of p53 and inhibition of caspase activity, REGγ is capable of inhibiting apoptosis.

### 3.2. Aging & Neurodegenerative Disease

Recent studies have indicated a relationship between REGγ and both aging and neurodegenerative disease, particularly in conditions of REGγ deficiency/decline. Firstly, REGγ deficiency was found to cause premature aging in mice via the REGγ-CK1δ-Mdm2-p53 pathway, whereby lower levels of REGγ were associated with accumulation of CK1δ and p53, leading to premature aging [10]. Consistently, an RNA-seq comparison analysis between 40- and 70-year-old human cortexes showed that REGγ expression was reduced in aged human beings [45]. Interestingly, a microarray analysis of hippocampal CA1 regions from 31 postmortem AD patients found a nearly 4-fold reduction in REGγ expression compared to normal controls [46]. These data indicate not only that REGγ expression diminishes as individuals age, but also that this decrease may be linked with neurodegenerative disorders. Providing a potential explanation for the correlation between REGγ decline and brain disorders, researchers showed that REGγ knockout mice exhibited increased GSK-3β activity and experienced several cognitive deficiencies such as defective prepulse inhibition (PPI), decreased working memory, and disability in nest building. Since GSK-3β overexpression has been linked with CNS diseases such as schizophrenia, REGγ-mediated regulation of GSK-3β is a likely mechanism through which REGγ affects the CNS [24]. Consistently, inhibition of GSK-3β was sufficient to rescue the compromised PPI phenotypes and deficiency in working memory. In another study of Huntington’s Disease (HD), patients were found to have reduced proteolytic activity in the brain and other tissues, leading to intraneuronal nuclear protein aggregates of mutant huntingtin. Interestingly, overexpression of REGγ was sufficient to rescue proteasome function in HD cells. At the same time, REGγ could improve cell viability in mutant-huntingtin expressing striatal neurons in the presence of pathological stressors such as quinolinic acid and MG132, a reversible proteasome inhibitor [47]. Similarly, a later study found that injecting lenti-REGγ virus into the striatum of mutant huntingtin-expressing mice improved motor control and helped to restore proteolytic activity to the UPS [48]. Thus, a decline in REGγ can contribute to both aging and neurodegenerative disorders via the pathways that lead to the accumulation of p53, tau, CK1δ, and GSK-3β.

### 3.3. DNA Damage Repair & Chromosomal Stability

REGγ is also an important factor in both DNA damage repair and chromosomal stability, such that its deficiency could lead to an increased likelihood of cell death during or after cell division due to slower repair of DNA damage, defective spindle structures, and aneuploidy. Firstly, REGγ is involved in double strand break (DSB) repair, as reduced REGγ levels result in longer repair times and more DNA damage hallmarks in several human cell lines. Mechanistically, REGγ was found to be rapidly localized to the site of DNA damage by ataxia-telangiectasia mutated (ATM) protein kinase and to recruit the 20S proteasome to the damaged site for efficient repair [49]. Supporting these results, a recent study on the involvement of lens epithelium-derived growth factor (LEDGF/p75), a transcriptional coactivator involved in DSB repair, on DNA damage repair found that LEDGF-depleted cells exhibit decreased REGγ protein levels and persistent activation of DNA damage signals such as γH2AX and BRCA1, indicating that the REGγ-proteasome likely plays a role in degrading molecules involved in DNA damage response [50]. Additionally, REGγ has been found to localize on chromosomes during the telophase and regulate spindle integrity, independent of the 20S proteasome. In this study, when cells were treated with the spindle damaging agent nocodazole, REGγ overexpression weakened mitotic arrest to trigger premature exit from mitosis, whereas REGγ underexpression exhibited the opposite effect. Furthermore, REGγ (−/−) mice and human fibroblasts with depleted expression of REGγ exhibited a marked aneuploidy and an increased frequency of abnormal metaphases, suggesting REGγ’s role in maintaining chromosomal stability [6]. In addition to its role in regulating chromosomal stability during mitosis, REGγ also appears to control the compaction of chromatin in a manner not dependent on binding with the 20S proteasome. In this study on a human cell line, FLIM-FRET microscopy analysis revealed that REGγ depletion correlates with chromatin decompaction, likely through REGγ-mediated maintenance of histone modifications H3K9me3 and H4K20me3 [7]. As such, REGγ is capable of regulating DNA damage repair, mitotic spindle integrity, and chromosomal compaction to maintain genomic stability.

Thus, based on loss-of-function studies, REGγ deficiency leads to cell death through various pathways. Firstly, REGγ prevents apoptosis through the downregulation of p53 and caspase such that its deficiency most likely leads to cell death. Furthermore, reduction of REGγ levels such as that which occurs with aging greatly facilitates p53/CK1δ/tau accumulation and GSK-3β overexpression, further leading to neurodegenerative disorders. In addition, REGγ deficiency leads to chromosomal instability and generates significant defects in DNA damage repair, which markedly reduces the viability of both cells and organisms. Therefore, REGγ is a key regulator of cell death processes.

## 4. Regulation of the Regulator

The evidence described above establishes REGγ as a regulator of various processes involved in both the life and death of cells. To carry out its specific functions, the expression and distribution of REGγ are manipulated at various levels, which has been revealed in the recent studies shown henceforth.

### 4.1. Transcriptional Regulation

As it relates to the transcriptional regulation of REGγ, p53/TGF-β signaling has been found to inhibit REGγ expression by Smad-dependent interaction with the REGγ promoter region. In this form of repression, p53 binds to the p53 response element (p53RE), a DNA binding domain in the REGγ promoter region. The activated TGF-β pathway triggers a p53-Smad3 inhibitory complex, followed by the formation of a Smad3/Nuclear receptor co-repressor 1 (N-CoR) complex for the repression of REGγ transcription. Mutant p53 was still able to bind to the p53RE region but prevented the formation of the Smad3/N-CoR complex. By doing so, mutant p53 can enhance the transcription of REGγ via prevention of inhibition, thus acting as an oncogene and contributing to cancer development [51]. In another study on endometrial cancer (EC), mutant p53-R248Q, the second most common p53 ‘hot spot’ mutation, was found to upregulate the expression of REGγ, as increased levels of mutant p53 correlated with increased levels of REGγ. Ultimately, this mutant p53-REGγ oncogenic pathway contributed to the evolution of EC [52]. Additionally, a genomic sequence analysis of a Drosophila cell line revealed that the REGγ promoter region contains transcription regulatory elements which often appear in the promoters of DNA replication or cell cycle progression genes. This indicates that expression of cell cycle regulatory proteins would be correlated with REGγ expression [27]. Thus, REGγ transcription is regulated by the p53/TGF-β signaling cascade and other cell cycle-regulatory factors.

### 4.2. Post-transcriptional Regulation

At the post-transcriptional level, miR-7-5p was found to bind to the REGγ 3’UTR for reduction of both mRNA and protein levels. In a breast cancer cell line, activation of miR-7-5p signaling inhibits cell proliferation, leading to apoptosis. Expectedly, introduction of an miR-7-5p inhibitor resulted in increased REGγ protein levels [43], and Cerebellar degeneration-related protein 1 antisense RNA (CDR1as), a circular RNA involved in the inhibition of miR-7, led to upregulation of REGγ in a breast cancer cell line [53]. Confirming these results, a study on gastric cancer cells found that downregulation of CDR1as promoted the cytotoxic effects of a traditional cancer drug Diosbulbin-B by inhibiting REGγ via the upregulation of miR-7-5p [54]. Similarly, miR-195-5p was also found to inhibit REGγ, as an miR-195-5p inhibitor prevents apoptosis and increases cell growth by preventing the inhibition of REGγ [40]. Therefore, REGγ is downregulated at the post-transcriptional level via miRNA interference.

### 4.3. Post-translational Regulation

Interestingly, a recent study found that NEFA-interacting nuclear protein 30 (NIP30), a negative regulator of REGγ, binds directly to REGγ for its inhibition. Activation of NIP30 attenuates cancer cell growth and sensitizes p53-compromised cells to chemotherapy. The study also showed that p21 protein levels were upregulated in the presence of NIP30 but p21 mRNA levels were unaffected, indicating REGγ’s inability to degrade p21 in the presence of NIP30. Cell division cycle 25A (CDC25A), a key cell cycle regulatory phosphatase that is degraded in response to DNA damage, was found to dephosphorylate NIP30 for its inactivation, thus preventing it from binding to REGγ. DNA damage by UV radiation reduced CDC25A levels sharply, which results in increased NIP30 phosphorylation, leading to inhibition of p21 degradation. Thus, a CDC25A-NIP30-REGγ regulatory axis can be established [55]. Similarly, another recent study on proteasomal inhibition in multiple myeloma (MM) cells found that indirubine-3’-monoxime (I3MO), a derivative of indirubin, significantly suppresses the growth of MM cells by directly binding to and inhibiting REGγ. This study also showed that cells resistant to bortezomib, an inhibitor of the 20S catalytic core particle, could be sensitized to bortezomib-induced apoptosis upon introduction of I3MO and subsequent REGγ inhibition [56].

In addition to regulation by NIP30 and I3MO, REGγ can be SUMOylated at multiple sites by SUMO-1, SUMO-2, and SUMO-3 to control its distribution and stability in the cell. In SUMOylation-deficient cells, REGγ was found to have an attenuated ability to degrade p21, suggesting the functional role of SUMOylation for both improving REGγ-mediated degradation and allowing it to target a broader range of substrates [57]. Additionally, it was found that the REGγ-proteasome complex degrades proteins such as p21 and HCV core protein more rapidly in an oxidative environment, and antioxidants counteract this oxidation-induced protein degradation. The addition of MG132, a proteasome inhibitor, or silencing of REGγ were both able to block this oxidant-induced degradation of p21. Hence, REGγ activity can be regulated through the manipulation of the oxidative state of the surrounding cellular environment [58]. In summary, REGγ can be regulated post-transcriptionally by manipulation of NIP30, CDC25A, SUMOylation, and modification of the oxidative state of the cellular environment to control the degradative ability of the REGγ-proteasome complex.

REGγ is transcriptionally regulated by p53-Smad3-dependent repression, mutant p53-mediated upregulation, and transcription factors functioning in cell cycle progression. After transcription, miR-7-5p and miR-195-5p are able to target REGγ for inhibition, and the addition of miRNA inhibitors such as CDR1as can prevent this interference. Lastly, REGγ can be regulated post-translationally with SUMOylation, NIP30 binding, CDC25A-mediated deactivation of NIP30, or alteration of the oxidative state of the cellular environment to control the distribution of REGγ and the degradative capacity of the REGγ-proteasome complex.

## 5. Conclusion and Future Directions

**Figure 2:**
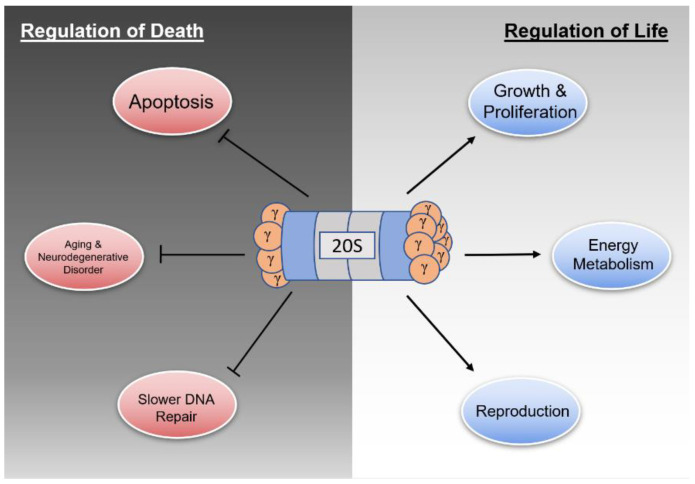
Regulatory functions of the REGγ-proteasome. Under normal expression conditions, REGγ maintains homeostasis via maintenance of processes listed under Regulation of Life and downregulation of processes listed under Regulation of Death.

As shown previously, REGγ plays a decisive role in maintaining the homeostasis of the cellular proteome (Figure 2). Given the facts that REGγ regulates cellular health in a plethora of ways and that mutations in REGγ are rarely discovered, dysregulation affecting the expression of REGγ inevitably carries significant consequences for both cellular and organismal well-being. 

With research having uncovered various new pathways for the regulation of REGγ at the transcriptional, post-transcriptional, and post-translational levels, manipulation of REGγ expression via therapeutic targeting becomes a viable and important direction for future research. Despite numerous studies, data regarding both the structure and substrate targeting mechanisms of REGγ are still lacking. In order to develop a foundation of knowledge for future studies, it will be necessary to first conduct structural analysis of the REGγ-proteasome in complexes with substrates in order to reveal the mechanistic details surrounding substrate binding and degradation. These data could help to elucidate the specific chemical and physical properties that facilitate REGγ-mediated protein degradation. Such studies focusing on the mechanism of substrate recognition by REGγ will facilitate the discovery of additional substrates and their functions. Furthermore, with research having already identified REGγ as a potential drug target by using NIP30 inhibition, crystallographic and electron microscopic analysis of the REGγ-proteasome-NIP30 complex could reveal new inhibitory mechanisms. Given this information, new inhibitory compounds could be engineered to safely treat patients with drug-resistant cancer. 

## Data Availability

Not applicable.
